# The impact of on‐field rehabilitation on return to play and ACL re‐injury risk after ACL reconstruction in football (soccer) players: A study on 401 consecutive cases

**DOI:** 10.1002/ksa.70392

**Published:** 2026-04-14

**Authors:** Francesco Della Villa, Filippo Picinini, Stefano Di Paolo, Alberto Scavone, Daniele Caminati, Jacopo Gamberini, Matthew Buckthorpe

**Affiliations:** ^1^ Isokinetic Medical Group, Education and Research Department FIFA Medical Centre of Excellence Bologna Italy; ^2^ Isokinetic Medical Group FIFA Medical Centre of Excellence London UK; ^3^ Faculty of Sport, Technology and Health Sciences St Mary's University, Twickenham London UK

**Keywords:** ACL injury, context, injury aetiology, injury prevention, timing

## Abstract

**Purpose:**

Examine the association between preoperative, intraoperative and postoperative variables, including on‐field rehabilitation (OFR) participation and return to play (RTP) rates and re‐injury risk in a large cohort of 11‐a‐side football (soccer) players after anterior cruciate ligament reconstruction (ACLR).

**Methods:**

Data from 401 male football players who underwent primary ACLR were retrospectively analysed. All players completed a standardised rehabilitation protocol, involving a period of OFR. Participants were stratified by competitive level (professionals and amateurs), and between‐group differences in RTP and re‐injury outcomes were documented. The association between preoperative, intraoperative and postoperative variables for each outcome was assessed using logistic regressions, controlling for competitive level.

**Results:**

Median follow‐up time was 40.6 months post‐ACLR. Eighty‐four percent of players RTP at their pre‐injury competitive level, with professionals (88%) and amateurs (83%) returning in 5.9 ± 2.1 and 6.9 ± 3.2 months, respectively. Greater OFR volume (odds ratio [OR], 1.06; 95% confidence interval [CI], 1.00–1.12; *p* = 0.034) and weekly frequency (OR, 1.53; 95% CI, 1.00–1.07; *p* = 0.014) were associated with increased RTP likelihood. High OFR compliance was associated with higher odds of RTP (OR = 2.62, *p* = 0.003), in which 91% of compliant players RTP at their pre‐injury competitive level. Forty‐two players (10%) sustained a second ACL injury (20 ipsilateral and 22 contralateral). OFR variables were not significantly associated with overall second ACL re‐injury risk. In an exploratory subgroup analysis of young (<20 years old) players, OFR compliance was associated with lower odds of ipsilateral re‐injury (OR = 0.23, *p* = 0.041).

**Conclusion:**

ACLR football players in our cohort had high RTP (88%) and low ACL re‐injury risk (10%). Greater exposure to OFR, particularly higher volume and weekly frequency, was associated with an increased likelihood of RTP. No association was observed between OFR compliance and overall second ACL injury risk, although in young players, greater OFR compliance was associated with a reduction in ipsilateral ACL re‐injury.

**Level of Evidence:**

Level IV.

**Abbreviations**
ACL: anterior cruciate ligament
ACLR: anterior cruciate ligament reconstruction
BMI: body mass index
BPTB: bone–patellar tendon–bone
CI: confidence intervals
cm: centimetre
FIFA: Fédération Internationale de Football Association
HT: hamstring tendon
IKDC: International Knee Documentation Committee
IQR: interquartile range
kg: kilogram
LCL: lateral collateral ligament
m: metre
MCL: medical collateral ligament
MRI: magnetic resonance imaging
*N*
: number
OFR: on‐field rehabilitation
OR: odds ratio
*Q*–*Q*
: quantile–quantile
QT: quadriceps tendon
RTP: return to play
SD: standard deviation

## INTRODUCTION

Anterior cruciate ligament (ACL) injury has devastating consequences and results in prolonged absences from football (soccer) [6, 23, 33, 41, 51]. While most professional players return to play (RTP) at high rates (97%) within 12 months after ACL reconstruction (ACLR) [51], amateurs have much lower return to sport (RTS) rates, with 81% resuming some form of sporting activity and only 55%–65% returning to pre‐injury sporting levels [2, 4, 28]. Moreover, nearly one in five professional players and one in three young amateur players (age < 25 years old) sustain a second ACL injury following RTP [23, 44, 51, 52]. As such, there is a need to enhance these outcomes after ACLR.

A criteria‐based approach with transition through rehabilitation stages [14, 15, 19], and subsequent RTS continuum [6, 18], has been advocated to improve RTP rates. Buckthorpe et al. [18] updated the RTS continuum to include structured progressions of on‐field rehabilitation (OFR), return to team training (RTT), return to competitive match play and finally return to performance. OFR is considered the vital bridge between the rehabilitation stages, often involving medically supervised in‐clinic rehabilitation and return to the team environment [16, 17, 18, 37]. A structured OFR programme has been shown to improve football aspects of players' physical preparedness (strength, cardiovascular fitness) for RTP after ACLR [12, 24] and to reduce RTP times and re‐injury rates following a musculoskeletal injury [49]. Although recent research documenting OFR workloads in football players after primary ACLR has been published [45], the specific impact of OFR participation on RTP outcomes and re‐injury risk in football players after ACLR remains unclear [7, 8].

This study aimed to evaluate the effect of OFR on RTP outcomes and second ACL injury risk in a large cohort of 11‐a‐side football players across different levels of play. It was hypothesised that players would demonstrate (i) a higher RTP rate at the same level and (ii) a lower second ACL injury rate compared to the current literature.

## METHODS

### Participants, inclusion and exclusion criteria

**Figure 1:**
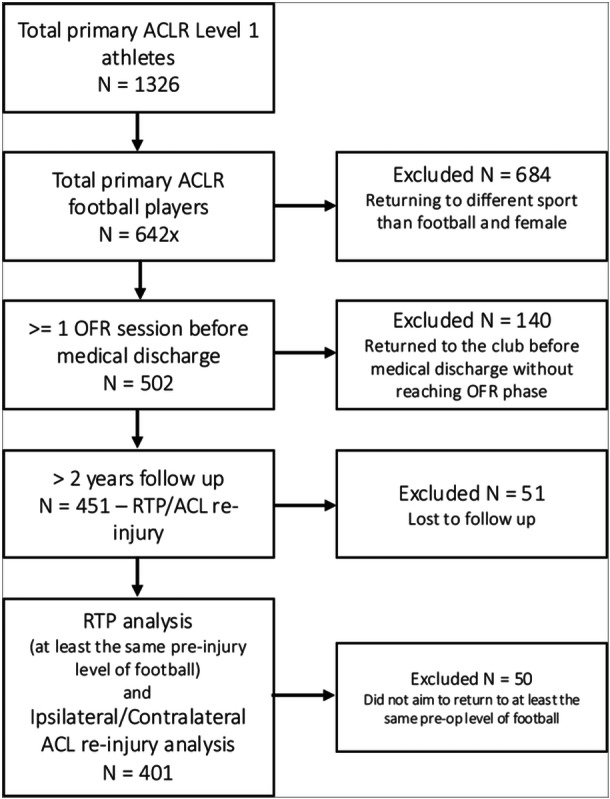
Flowchart of participant inclusion in the analysis. ACL, anterior cruciate ligament; ACLR, anterior cruciate ligament reconstruction; OFR, on‐field rehabilitation; RTP, return to play.

Four hundred and one football players undertaking rehabilitation with the ambitions to return to at least the same pre‐injury competitive level of football (organised competitive matches played on standard‐sized pitches, adhering to the Fédération Internationale de Football Association (FIFA) game's rules, within professional or amateur leagues), following primary ACLR, were included in this study (see Table 1). Participants were retrospectively selected from a sample dataset of 1326 consecutive primary ACLRs performed between 2010 and 2014, with athletes from various sports and competition levels (Figure 1). Players attended a structured and criteria‐based rehabilitation programme at Isokinetic Medical Group (FIFA Medical Centre of Excellence) in Bologna, Italy. Additional inclusion criteria consisted of (1) clearance from the surgeon and sports medicine physician to RTP, (2) pre‐surgical intention to return to competitive football, (3) completion of at least one OFR session before medical discharge and (4) >2 years follow‐up. Players with revision ACLR or those undergoing concurrent repair/reconstruction of other knee ligaments were excluded. To be clear, only male players were included in this study. Football is played by millions of women around the world, and the *Knee Surgery, Sports Traumatology and Arthroscopy* (*KSSTA*) journal encourages research that includes gender‐based analysis. The number of female players who met the inclusion criteria was small (*n* = 12). Additional research is needed on female players to fill this gap. Our group is going towards this direction. All participants provided informed consent when entering Isokinetic for rehabilitation. The study received ethical approval from the Bioethical Committee of the University of Bologna (no. 0007503 of 8 January 2025).

**Table 1:** Players' information, type of ACL injury, mechanism of ACL injury and graft choice for ACL reconstruction.

	*n* (%) or mean ± SD			Professional‐Amateur
Measurements	All players (*n* = 401)	Professional (*n* = 42)	Amateur (*n* = 359)	*p* value
Pre‐operative variables				
Age (years)	26.0 ± 8.5	23.1 ± 4.6	26.3 ± 8.7	**<0.001**
Height (cm)	178 ± 6.3	182 ± 5.3 182 (180–184)^a^	178 ± 6.3 178 (178–179)^a^	0.346
Body mass (kg)	75.1 ± 8.6	75.6 ± 4.9	75.0 ± 8.9	**0.001**
Body mass index (BMI) (kg/m^2^)	23.5 ± 2.2	22.8 ± 1.2	23.6 ± 2.3	**<0.001**
Pre‐injury Tegner score	8.4 ± 0.9	9.5 ± 0.6	8.3 ± 0.9	**<0.001**
ACL injury mechanism				
Direct contact	63 (16)	10 (24)	53 (15)	—
Indirect contact	65 (16)	6 (14)	59 (16)	—
Non‐contact	273 (68)	26 (62)	247 (69)	—
Time from injury to surgery (days)	41.4 ± 43.1	22.7 ± 19.8	55.0 ± 58.8	**0.013**
Intra‐operative variables				
ACL graft type				
Autograft BPTB, *n* (%)	50 (12)	6 (14)	44 (12)	—
Autograft HT, *n* (%)	347 (87)	34 (81)	313 (87)	—
Autograft QT, *n* (%)	2 (0.5)	0 (0)	2 (0.6)	—
Allograft, *n* (%)	2 (0.5)	2 (5)	0 (0)	—
Medial meniscus injury				
Nil	302 (75)	34 (81)	268 (75)	—
Meniscectomy	79 (20)	4 (10)	75 (21)	—
Repair	20 (5)	4 (10)	16 (4)	—
Lateral meniscus injury				
Nil	299 (75)	30 (72)	269 (75)	—
Meniscectomy	82 (20)	8 (30)	74 (21)	—
Repair	20 (5)	4 (10)	16 (4)	—
Medial collateral ligament injury				
Nil	357 (89)	37 (88)	321 (89)	—
Grades 1–2	36 (9)	3 (7)	33 (9)	—
Grade 3	8 (2)	2 (5)	5 (1)	—
Lateral collateral ligament injury				
Nil	388 (97)	41 (98)	347 (97)	—
Grades 1–2	11 (2)	0 (15)	11 (16)	—
Grade 3	2 (0.5)	1 (2)	1 (0.3)	—

*Note*: Values other than the number of participants are expressed as mean ± SD except where the data were non‐normally distributed, where these data are presented as median and IQR. Bold indicates statistically significant differences. Independent‐sample *t* tests were used for between‐groups comparison, with a significant difference (*p* < 0.05).

Abbreviations: ACL, anterior cruciate ligament; ACLR, anterior cruciate ligament reconstruction; BPTB, bone–patellar tendon–bone; cm, centimetre; HT, hamstring tendon; IQR, interquartile range; kg, kilogram; OFR, on‐field rehabilitation; QT, quadriceps tendon; SD, standard deviation.

a Non‐normally distributed data. All participants were male.

### The isokinetic rehabilitation approach

Players followed a structured rehabilitation framework delivered by a multidisciplinary team to optimise recovery involving five phases: (1) resolution of pain and swelling, (2) resolution of knee joint range of motion and flexibility, (3) restoration of muscle strength, (4) recovery of coordination and (5) sport‐specific retraining [20, 24, 25]. Rehabilitation activities were carried out across three different environments: the gym, the swimming pool and the football field.

The criteria for starting OFR were as follows [25]: clearance from the Sports Medicine Physician case manager; no knee ligament instability on clinical tests (Lachman, anterior drawer, pivot shift); no giving‐way episodes during the preceding phases; minimal pain (visual analogue scale <3 out of 10); absence or minimal effusion (Grade 0 or 0/1+); complete or nearly complete ROM (full extension, < 10° flexion deficit vs contralateral limb); an isokinetic maximal peak torque deficit of less than 20% between limbs [34, 36] and able to run on the treadmill at 8 km·h^−1^ per hour for >10 min [47].

Players commenced a five‐staged OFR programme, incorporating drills that replicated competitive football players' physical, technical and tactical game elements within a controlled setting [25] (see Supporting Information S1: Table 1). Progression through stages was based on the player's knee and soft tissue response to each session. Each 90‐min session was delivered on a regular 11‐a‐side outdoor grass pitch (100 × 50 m) by rehabilitation coaches with substantial experience rehabbing players from various football‐related injuries.

### Player's data

Collected data during the observational period included demographic, pre‐operative (e.g., age, body mass, height, body mass index [BMI], pre‐injury Tegner scale, level of football, ACL injury mechanism and time from injury to ACLR surgery), intra‐operative (e.g., ACL graft type, medial and lateral meniscal injury status, medical collateral ligament [MCL] injury, MCL grade, lateral collateral ligament [LCL] injury, LCL grade) and post‐operative information on each player (e.g., time from surgery to rehab, indoor rehab volume, time to OFR from surgery, OFR volume, overall rehab volume, number of OFR sessions, weekly OFR frequency). Data were organised into a custom‐developed anonymised Microsoft Excel sheet (Version 16.66.1; Microsoft) for further inspection and analysis.

### Follow‐up

Follow‐ups were conducted to determine players' RTP outcomes. Players were contacted via email and telephone to complete a questionnaire about their RTP outcomes, following the provision of informed consent. At follow‐up, participants completed the International Knee Documentation Committee (IKDC) questionnaire and were asked questions about their RTP outcomes, reasons for not returning to football, if they did not, pre‐injury and post‐injury levels of football, and whether they had sustained an ACL re‐injury. Participants who experienced a second ACL injury to either knee were identified at follow‐up, or if they returned to the clinic for management before that time point, a diagnosis of ipsilateral or contralateral ACL injury was confirmed with a magnetic resonance imaging (MRI) scan. The follow‐up questionnaire is included within the supplementary material (Supporting Information S1: Appendix 1).

### Statistical analysis

Demographic, pre‐operative, intra‐operative, post‐operative and follow‐up data were summarised using descriptive statistics and presented per level of play. Continuous variables were reported as mean ± standard deviation (SD) or median (range), depending on distribution assessed via quantile–quantile (*Q*–*Q*) plots. Categorical variables were presented as counts and percentages. Group differences in continuous variables were evaluated using independent samples *t* tests, with Levene's test assessing equality of variances. Chi‐square tests (*χ* ^2^) assessed associations between categorical variables, with odds ratios (ORs) and 95% confidence intervals (CIs) calculated when significant.

Binary logistic regression models were fitted to predict the likelihood of RTP to at least the same pre‐injury level and second ACL injury of the observed cohort. Collinearity diagnostics (linear regression analysis with collinearity diagnostics) indicated multicollinearity between the number of indoor rehab sessions and weekly indoor rehab frequency, with Variance Inflation Factor values exceeding 10 and Tolerance values < 0.10; therefore, they were removed from the final models. A stepwise‐forward selection (*p* = 0.01 in, *p* = 0.05 out) identified significant predictors across pre‐operative, intra‐operative and post‐operative variables. Kaplan–Meier survival curves were used to visualize the probabilities of time‐to‐RTP (months) and time‐to‐ACL re‐injury (months) according to the outcomes of the logistic regression models. Log‐rank tests assessed differences between survival curves. All statistical analyses were conducted in SPSS (Version 29; IBM Corporation), with statistical significance at *p* < 0.05.

## RESULTS

### Demographics, surgery and field data

#### Pre‐, intra‐ and post‐operative data

Professionals were significantly younger and had lower BMI than amateurs (*p* < 0.001, Tables 1 and 2). Non‐contact was the primary ACL injury mechanism (68%), while direct contact injuries were more common in professionals than amateurs (24% vs. 15%). Hamstring tendon autograft was the predominant graft choice in ACLR (87%), with no significant differences in graft type or meniscal and collateral ligament injuries between groups. Time from injury to surgery was shorter for professionals (22.7 ± 19.8 days vs. 55.0 ± 58.8 days; *p* = 0.013). The OFR phase commenced at 111 ± 42 days post‐surgery, with professionals completing significantly more sessions (25 ± 14 vs. 10 ± 6; *p* < 0.001) and at a higher weekly frequency (3.0 ± 1.3 vs. 1.7 ± 0.8; *p* < 0.001) than amateurs. No significant differences were noted between professional and amateur players for intra‐operative variables. The median follow‐up time was 40.6 (interquartile range [IQR] 1.5) months after ACLR (*n* = 401) (Table 2).

**Table 2:** Rehabilitation measurements and follow‐up outcomes.

	*n* (%) or mean ± SD			Professional‐Amateur
Measurements	All players (*n* = 401)	Professional (*n* = 42)	Amateur (*n* = 359)	*p* value
Post‐operative variables				
Time from surgery to rehabilitation (days)	24.0 ± 34.0	23.0 ± 43.6 23.0 (11.9–34.1)^a^	24.2 ± 33.6 24.2 (20.5–27.8)^a^	0.665
Time to OFR from surgery (days)	111 ± 42.3	101 ± 42.2 101 (88.2–115)^a^	112 ± 42.3 112 (108–116)^a^	0.994
OFR volume (days)	52.6 ± 30.8	63.0 ± 35.2 63.0 (52.8–73.2)^a^	51.4 ± 30.1 51.4 (48.0–54.8)^a^	0.279
Overall rehabilitation volume (days)	177 ± 58.5	174 ± 55.9 174 (155–193)^a^	177 ± 58.0 177 (171–184)^a^	0.643
Number of indoor rehabilitation sessions (*n*)	59.9 ± 37.6	118 ± 60.6	53.3 ± 27.3	**<0.001**
Weekly indoor rehabilitation frequency (*n*)	3.0 ± 1.7	6.0 ± 0.7	2.7 ± 1.2	**<0.001**
Number of OFR sessions (*n*)	12.0 ± 8.3	25.4 ± 14.3	10.2 ± 6.1	**<0.001**
Weekly OFR frequency (*n*)	1.8 ± 1.0	3.0 ± 1.3	1.7 ± 0.8	**<0.001**
Follow‐up outcomes				
Time to follow‐up (months)	42.2 ± 12.5	42.5 ± 13.9 42.5 (37.9–46.9)^a^	42.2 ± 12.3 42.2 (40.6–43.7)^a^	0.623
RTP outcomes: ‘Did you return to the same pre‐injury level of football?’				
Yes	335 (84)	37 (88)	298 (83)	—
No	66 (16)	5 (12)	61 (17)	—
Time to RTP (months)	6.8 ± 3.1	5.9 ± 2.1 5.9 (4.9–6.9)^a^	6.9 ± 3.2 6.9 (6.5–7.2)^a^	0.066
Second ACL injury: ‘Did you suffer a second ACL injury?’				
Ipsilateral ACL re‐injury				
Yes	20 (5)	0 (0)	20 (6)	—
No	381 (95)	42 (100)	339 (94)	—
Time to second ipsilateral ACL injury from surgery (months)	14.0 ± 9.9	—	14.0 ± 9.9	—
Contralateral ACL re‐injury				
Yes	22 (5)	3 (7)	19 (5)	—
No	379 (95)	39 (93)	340 (95)	—
Time to second contralateral ACL injury from surgery (months)	25.9 ± 12.5	27.3 ± 4.8	25.7 ± 13.3	0.638
IKDC at follow‐up	95.0 ± 6.8	96.2 ± 5.0	94.7 ± 7.0	**0.039**

*Note*: Values other than the number of participants are expressed as mean ± SD except where the data were non‐normally distributed, where these data are presented as median and IQR. Independent‐sample *t* tests were used for between‐groups comparison, with a significant difference (*p* < 0.05). Bold indicates statistically significant differences.

Abbreviations: ACL, anterior cruciate ligament; ACLR, anterior cruciate ligament reconstruction; BPTB, bone–patellar tendon–bone; cm, centimetres; HT, hamstring tendon; IKDC, International Knee Documentation Committee; IQR, interquartile range; kg, kilogram; OFR, on‐field rehabilitation; QT, quadriceps tendon; RTP, return to play; SD, standard deviation.

a Non‐normally distributed data. All participants were male.

### RTP rate

**Figure 2:**
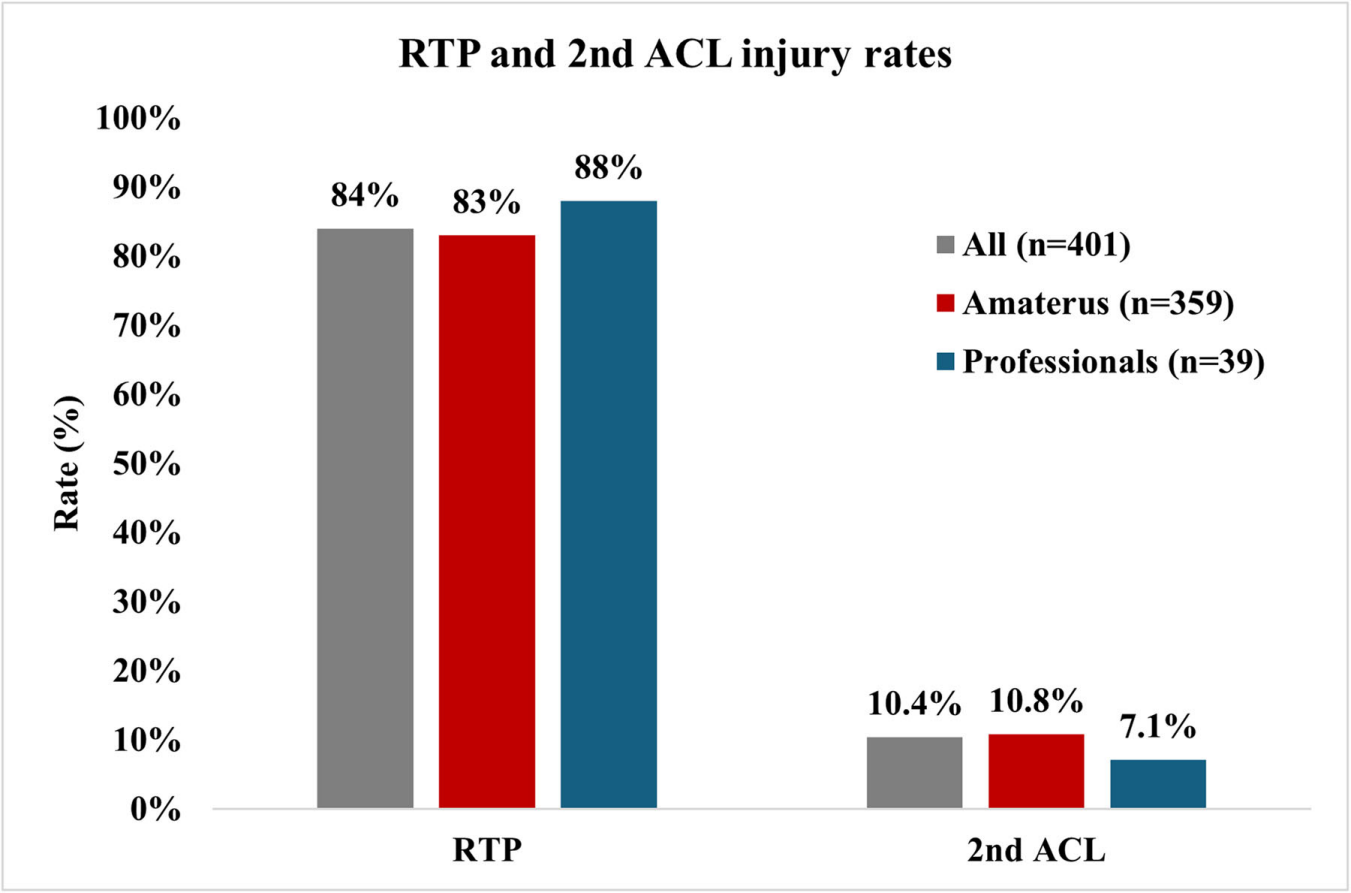
RTP (at previous level of competition) and second ACL injury rates according to players' level. ACL, anterior cruciate ligament; RTP, return to play.

All players resumed some form of football, with 84% returning to their pre‐injury competitive level. Professionals reported an 88% RTP rate in 5.9 ± 2.1 months, whilst 83% of amateurs RTP at 6.9 ± 3.2 months (*p* > 0.05) (see Figure 2).

The logistic regression model showed that OFR volume (OR = 1.06, *p* = 0.006) and weekly OFR frequency (OR = 1.53, *p* = 0.011) were significantly associated with greater RTP rate (see Table 3).

**Table 3:** Differences between RTP and no RTP, and time to RTP at follow‐up.

Measurements	RTP, *n* (%) or mean ± SD			RTP versus no RTP
	Yes	No	*p* value	OR: Yes RTP (95% CI)
Pre‐operative variables				
Age (years)	26.0 ± 8.6	25.8 ± 8.2	0.899	—
Level of football				
Professional players (*n* = 42)	37 (88)	5 (12)	0.400	—
Amateur players (*n* = 359)	298 (83)	61 (17)		
Pre‐injury Tegner score	8.4 ± 1.0	8.6 ± 0.8	0.223	—
ACL injury mechanism				
Direct contact (*n* = 63)	53 (84)	9 (16)	0.917	—
Indirect contact (*n* = 65)	55 (85)	11 (15)		
Non‐contact (*n* = 273)	227 (83)	46 (17)		
Time from injury to surgery (days)	33.4 ± 36.7	68.3 ± 36.9	0.445	—
Intra‐operative variables				
ACL graft type				
Autograft BPTB (*n* = 50)	44 (88)	6 (12)	0.419	—
Autograft HT (*n* = 347)	288 (83)	59 (17)		
Autograft QT (*n* = 2)	2 (100)	0 (0)		
Allograft (*n* = 2)	1 (50)	1 (50)		
Post‐operative variables				
Time from surgery to rehabilitation (days)	24.3 ± 35.8	24.1 ± 31.0	0.974	—
Time to OFR from surgery (days)	142 ± 55.5	141 ± 51.8	0.943	—
OFR volume (days)	52.7 ± 30.1	49.6 ± 32.4	0.482	—
Overall rehabilitation volume (days)	176 ± 59.1	183 ± 58.0	0.409	—
Time to RTP (days)	203 ± 90.2	227 ± 123	0.059	—
Number of indoor rehabilitation sessions (*n*)	61.8 ± 39.8	50.0 ± 21.8	0.076	—
Weekly indoor rehabilitation frequency (*n*)	3.1 ± 1.7	2.6 ± 1.3	0.082	—
Number of OFR sessions (*n*)	12.2 ± 9.0	9.0 ± 4.6	**0.006**	**1.06 (1.00–1.12)**
Weekly OFR frequency (*n*)	1.9 ± 1.0	1.5 ± 0.8	**0.011**	**1.53 (1.10–2.13)**
IKDC score at follow‐up	95.4 ± 6.2	92.2 ± 9.5	**<0.001**	**1.04 (1.00–1.07)**

*Note*: Bold values signifies statistical significance.

Abbreviations: ACL, anterior cruciate ligament; ACLR, anterior cruciate ligament reconstruction; BPTB, bone‐patellar tendon‐bone; CI, confidence interval; HR, hazards ratio; HT, hamstring tendon; IKDC, International Knee Documentation Committee; OFR, on‐field rehabilitation; OR, odds ratio; QT, quadriceps tendon; RTP, return to play; SD, standard deviation.

**Figure 3:**
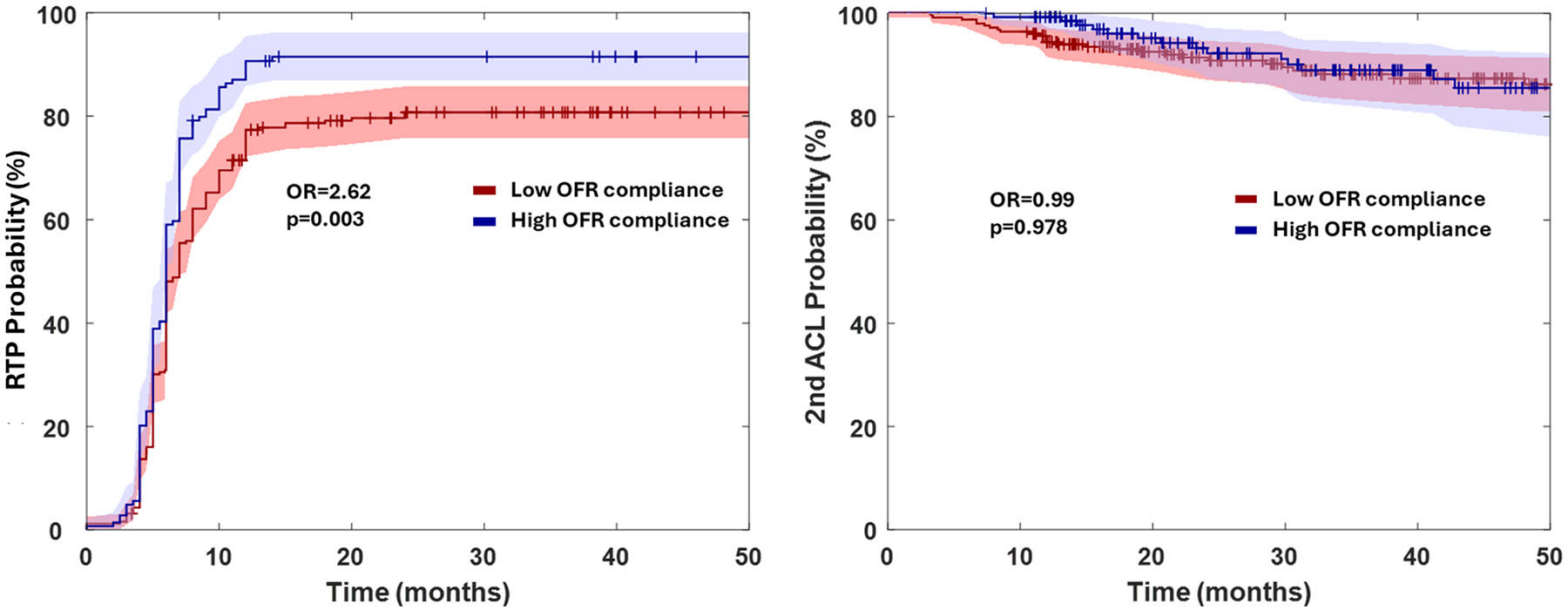
RTP probability (left) and survivorship from second ACL injury (right) in players with low (red) and high (blue) compliance to OFR, defined as *n* > 11 total sessions and weekly frequency *n* > 2 sessions/week. ACL, anterior cruciate ligament; OFR, on‐field rehabilitation; OR, odds ratio; RTP, return to play.

The combination of OFR volume and weekly OFR frequency was further investigated in a new metric defined as ‘OFR compliance’: players with both metrics above the mean (OFR volume >11 and OFR frequency >2) were defined as high compliance (*n* = 143), while any other combination of the two metrics was defined as low compliance (*n* = 254). An OR = 2.62 [1.37–4.99] for RTP was found in players with high OFR compliance (*p* = 0.003, Figure 3). OFR‐compliant players achieved a RTP rate of 91%.

### Second ACL injury

Forty‐two players (10.5%) experienced a second ACL injury, with 20 ipsilateral (5.0%) and 22 contralateral (5.5%) injuries. Ipsilateral re‐injuries occurred earlier after ACLR than contralateral injuries (14.0 ± 9.9 vs. 25.9 ± 12.5 months, *p* < 0.001). Professionals reported no ipsilateral re‐injuries and a similar rate of contralateral injuries (%) to amateurs (overall second ACL: 7.1% vs. 10.9%). Among the demographic features, lower age (OR = 0.91, *p* < 0.001) was associated with higher second ACL injury occurrence; the same effect was noted for ipsilateral (OR = 0.82, *p* < 0.001), but not contralateral injuries. Higher pre‐injury Tegner score was also associated with second ACL injury (OR = 1.57, *p* = 0.021, Table 4), and the same effect was noted in contralateral (OR = 2.01, *p* = 0.022), but not in ipsilateral injuries. Compliance in OFR was not associated with second ACL injury risk (OR = 0.99, *p* > 0.05, Figure 3).

**Table 4:** Differences between ACL re‐injury and no ACL re‐injury, and time to ACL re‐injury at follow‐up.

Measurements	ACL re‐injury, *n* (%) or mean ± SD			ACL re‐injury versus no ACL re‐injury
	Yes	No	*p* value	OR: Yes ACL re‐injury (95% CI)
Pre‐operative variables				
Age (years)	21.6 ± 7.5	26.8 ± 8.8	**<0.001**	**0.91 (0.85–0.98)**
Level of football				
Professional players (*n* = 42)	3 (7)	39 (93)	0.456	—
Amateur players (*n* = 359)	39 (11)	320 (89)		
Pre‐injury Tegner score	8.8 ± 0.6	8.4 ± 1.0	**0.021**	**1.57 (1.07–2.32)**
ACL injury mechanism				
Direct contact (*n* = 63)	5 (8)	58 (92)	0.766	—
Indirect contact (*n* = 65)	6 (9)	58 (89)		
Non‐contact (*n* = 273)	31 (11)	243 (89)		
Time from injury to surgery (days)	37.5 ± 28.9	60.6 ± 40.5	0.250	—
Intra‐operative variables				
ACL graft type				
Autograft BPTB (*n* = 50)	5 (10)	45 (90)	0.310	—
Autograft HT (*n* = 347)	36 (10)	311 (89)		
Autograft QT (*n* = 2)	0 (0)	2 (100)		
Allograft (*n* = 2)	1 (50)	1 (50)		
Post‐operative variables				
Time from surgery to rehabilitation (days)	30.9 ± 39.8	23.5 ± 34.4	0.193	—
Time to OFR from surgery (days)	101 ± 32.1	112 ± 43.2	0.106	—
OFR volume (days)	48.7 ± 26.8	52.6 ± 30.8	0.445	—
Overall rehabilitation volume (days)	165 ± 49.8	179 ± 59.8	0.151	—
Time to RTP (days)	184 ± 61.3	209 ± 99.8	0.113	—
Number of indoor rehabilitation sessions (*n*)	53.4 ± 27.8	60.6 ± 38.6	0.245	—
Weekly indoor rehabilitation frequency (*n*)	3.1 ± 1.5	3.0 ± 1.7	0.806	—
Number of OFR sessions (*n*)	12.0 ± 7.8	11.6 ± 8.6	0.761	—
Weekly OFR frequency (*n*)	1.9 ± 0.8	1.8 ± 1.0	0.761	—

*Note*: Bold values signifies statistical significance.

Abbreviations: ACL, anterior cruciate ligament; ACLR, Anterior Cruciate Ligament reconstruction; BPTB, bone–patellar tendon–bone; CI, confidence interval; HR, hazards ratio; HT, Hamstring tendon; OR, odds ratio; OFR, on‐field rehabilitation; QT, quadriceps tendon; RTP, return to play; SD, standard deviation.

**Figure 4:**
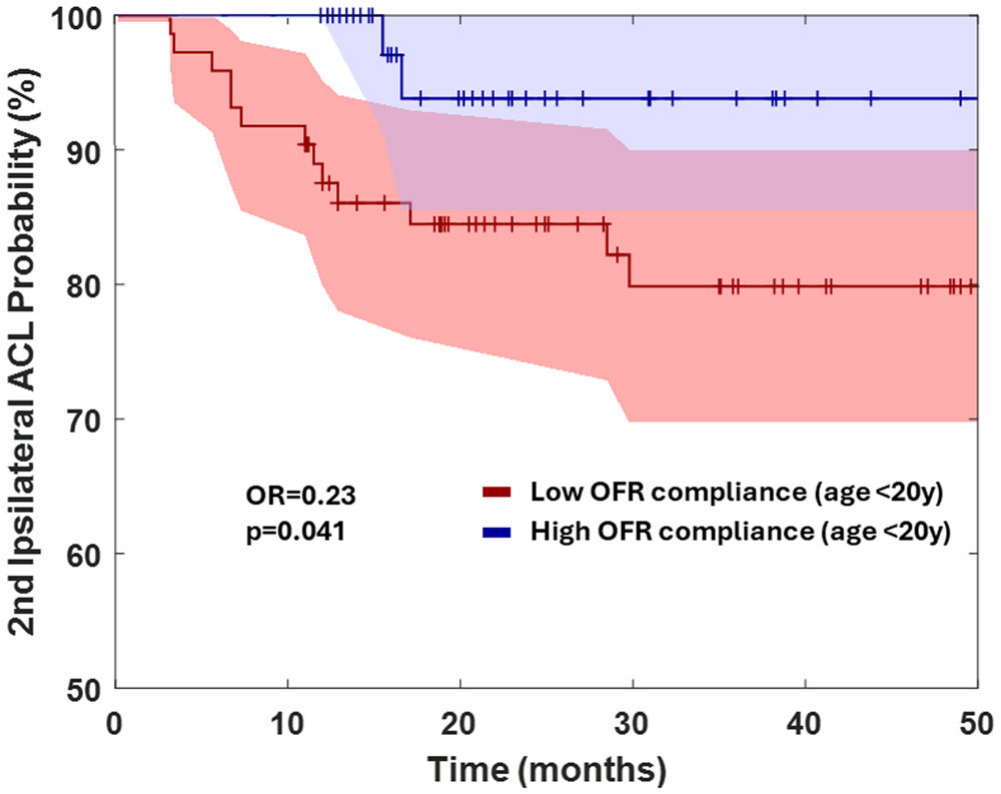
Survivorship from second ipsilateral ACL injury (graft rupture) in young players (*N* = 116, age < 20 years old) with low (red, *n* = 73) and high (blue, *n* = 43) compliance to OFR, defined as *n* > 11 total sessions and weekly frequency *n* > 2 sessions/week. ACL, anterior cruciate ligament; OFR, on‐field rehabilitation; OR, odds ratio.

In young players (academy age, <20 years old, *n* = 116), being highly compliant to OFR showed a protective effect (OR = 0.22 [0.05–1.05], *p* = 0.041, Figure 4) towards second ipsilateral re‐injuries (2/43 injuries in the compliant vs. 13/73 in the non‐compliant subgroups), while no significant effect was found for overall and contralateral second ACL injury (Figure 3).

## DISCUSSION

The main finding of this study was that ACLR football players adhering to structured criteria‐based OFR reported a high RTP rate (84%) with a relatively low second ACL injury rate (10%) at a minimum 2‐year follow‐up. Professional players reported superior outcomes than amateurs, with 88% RTP at the same level and 7% second ACL injury rates. Compliance in OFR, defined as a combination of both high OFR volume (*n* > 11 total sessions) and weekly frequency (*n* > 2 sessions/week), was associated with greater odds of RTP (OR = 2.62) in which 91% of compliant players RTP. OFR compliance was not associated with overall second ACL injury risk (OR = 0.99). In the sub‐group of players aged <20 years old, compliance was associated with lower odds of ipsilateral graft rupture (OR = 0.23).

### RTP rate

RTP rates at the same pre‐injury level (84%), particularly among amateurs (83%), were higher than those reported in systematic reviews of amateur football players after ACLR (65%–74%) [3, 5, 29], which is consistent with our first hypothesis. Professional players in our study achieved high RTP rates at their previous competitive level (88%), with comparable RTP times to previously published similar cohorts [33]. Our findings align with King et al. [30], who observed 81% RTP following primary ACLR in competitive Level 1 athletes. Professional players in our cohort also RTP earlier (5.9 ± 2.1 months) than amateurs, which may be explained by, among other factors, a greater number of rehabilitation sessions completed by professionals prior to medical discharge [28]. The higher RTP rates observed in professionals versus amateurs are likely a function of early diagnostics, referral to experienced surgeons, daily expert‐led rehabilitation and large financial motivation for the player to RTC [28, 51]. Amateurs in this study RTP at greater rate and earlier (6.9 ± 3.2 months) than findings from Manojlovic et al. [35], who reported a 53% RTP at the pre‐injury level, 8.7 months after ACLR.

The higher RTP rates observed in our cohort compared with previous reports may reflect differences in rehabilitation structure, including the use of structured, criteria‐based rehabilitation framework incorporating OFR prior to medical discharge. An association between the number of OFR sessions completed and RTP probability was observed. Each additional OFR session in the amateur group increased RTP probability by 6%, with a similar trend observed in professionals (7%), although statistical significance was only reached in the amateur subgroup, due to statistical power. These findings suggest that greater OFR exposure prior to medical discharge is associated with higher RTP probability. Alongside OFR volume, our study documented a 53% increase in RTP probability for each additional session per week of OFR, highlighting the potential relevance of OFR weekly frequency. The combination of high OFR volume and weekly frequency, termed ‘high compliance’, was associated with higher RTP probability (+160%). Given the low RTP rates of amateur players in previous published cohorts [3, 5, 29], this finding may have implications for optimising RTP strategies after ACLR for football players. OFR includes a structured programme of movement progressions, football‐specific actions, fitness reconditioning and progressive exposure to football‐specific workloads [16, 17]. When implemented in a structured rehabilitation programme, OFR has previously been shown to improve lower limb muscle strength, knee function, cardiovascular fitness and workload readiness [23, 24, 25, 45, 48]. While evidence supports the benefits of completing an OFR programme for football players' physical readiness, the psychophysiological adaptations during this rehabilitation period have not been fully explored.

IKDC scores were also associated with RTP rates, with each unit increasing RTP odds by 4%. Higher IKDC scores at follow‐up have previously been associated with superior knee function, psychological readiness and increased likelihood of RTP at the same pre‐injury level in competitive athletes after ACLR [38, 40, 42, 50]. To our knowledge, this is the first study to examine this relationship in a large cohort of football‐only ACLR players, differentiating IKDC scores between those who did RTP and those who did not [35].

### Second ACL injury

The 11% ACL re‐injury rate among amateurs was lower than the 30% reported in previous studies with similar follow‐up times [44, 52] and comparable to rates of 12%–16% in other cohorts [13, 32, 53]. Consistent with prior findings, re‐injury rates in amateurs were similar for ipsilateral grafts (6%) and contralateral injuries (5%), with ipsilateral re‐injuries occurring earlier after ACLR [30]. In contrast, professionals experienced only contralateral re‐injuries (7%), aligning with data from elite‐level players [23]. The number of completed OFR sessions was not statistically associated with re‐injury risk. Likewise, OFR compliance was not associated with reduced ACL re‐injury risk. Considering that most ACL re‐injuries in football occur within 2 years after ACLR, in both amateurs [44, 52, 53] and professionals [51] the volume of OFR sessions may have a diminishing association with re‐injury risk over time, given that few ACL re‐injuries occur before RTP [23, 51]. The elevated risk of ACL re‐injury may be influenced by modifiable (e.g., neuromuscular control deficits) and non‐modifiable factors (e.g., genetic predisposition, anatomic variations) [11, 26], which we did not consider in this study. Future research should investigate whether OFR volume and activities (e.g., training load metrics) influence ACL re‐injury risk and other injuries, such as meniscal and lower limb muscle injuries.

While OFR compliance was not associated with overall second ACL injury risk in the full cohort, an association was observed between OFR compliance and lower odds (OR, 0.23) of ipsilateral graft rupture in players <20 years. Given the relatively small size of this subgroup, this finding should be interpreted cautiously and considered exploratory. Previous evidence has identified younger age as a key risk factor for second ACL injury in youth athletes participating in pivoting sports [27, 44, 53]. Moreover, graft rupture and other knee injuries are particularly frequent within the first year after RTP, in both amateurs and professionals [23, 27, 32, 53]. The present findings, therefore, warrant further investigation in larger prospective cohorts to determine whether OFR exposure influences re‐injury risk in younger football players.

Time to RTP was not associated with ACL re‐injury in either group, which is consistent with some evidence [30, 31]. Other research, with low‐certainty evidence, suggests earlier RTP may be associated with increased re‐injury risk in amateur players, but not professionals [46]. To our knowledge, our work is the first to explore the relationship between the timing of OFR commencement and, thus, completion of indoor rehabilitation and attainment of OFR entry criteria and re‐injury risk. Individualised rehabilitation timelines and cautious progression to high‐intensity football activities are warranted. The optimal OFR timing and RTP criteria to minimise re‐injury risk remain to be defined.

A higher pre‐injury Tegner score was associated with an increased risk of sustaining a second ACL injury, with the effect observed for contralateral, not ipsilateral, re‐injuries. Prior research has documented that young, high‐level athletes with a pre‐injury Tegner score ≥7 had nearly four‐fold higher odds of graft rupture [22, 53]. This evidence has been derived from mixed‐sport cohorts, making the current study the first to document this relationship in a homogeneous group of football players.

### Limitations

This is the first study to examine the role of OFR activity on RTS outcomes in a large (*n* = 401), homogeneous group of competitive football‐only players. While the findings contribute to understanding RTP outcomes in this population, the retrospective design precludes causal inference regarding the role of OFR in influencing these outcomes. Despite high RTP rates at the same pre‐injury competitive level in both groups, it remains unclear whether the players who returned to football achieved their pre‐injury performance levels, an important factor highlighted in previous research as essential for defining successful RTP outcomes [10, 35, 40]. Future research should incorporate objective match performance metrics and psychological measures to better characterise after ACLR recovery in football players. Training volume and intensity following OFR were not available, and activities during team‐based training were not monitored. As such, workload exposure during later phases of the RTS continuum could not be accounted for, limiting interpretation of re‐injury findings [18].

All participants were medically cleared for RTP by a surgeon and sports medicine physician; however, surgeon‐specific RTP criteria were unavailable due to the involvement of multiple operating surgeons. Standardised or surgeon‐specific clearance criteria would improve contextualisation of RTP decision‐making in future studies. Furthermore, whilst the sports medicine physician may have medically discharged the patients, this does not mean they necessarily completed the programme or achieved desired milestones. Players occasionally completed the rehabilitation process with the team or stopped rehabilitation early. This cohort was a homogenous cohort of male‐only football players treated at an Italian privately owned FIFA medical centre of excellence. As such, although these findings likely have wider implications beyond the cohort, generalisability to other sports, females [43] and rehabilitation settings and healthcare systems cannot be assumed. Given the findings of this work and potential value of OFR on outcomes after ACLR, further research exploring the effect of OFR is warranted in other cohorts. The number of academy‐age players (<20 years old) was a relatively small portion of the original sample size (116/401 players). Thus, results should be interpreted and generalised with caution. Furthermore, the patients partook in and completed their rehabilitation between 2010 and 2014. There have been considerable changes in ACLR surgery and rehabilitation and RTS practices after ACLR over the last 10–15 years. Our group has published many papers on the topic [14, 15, 16, 17, 18, 19, 20, 21], including innovations in late‐stage rehabilitation and RTS training, such as a greater focus on strength and power and restoration and profiling, fitness reconditioning, movement retraining and qualitative assessment and updates on OFR processes and practices. Equally, the physical demands of football, especially the high‐intensity actions (accelerations, decelerations, sprints), have increased dramatically over the last 15 years [1, 9, 39], raising the fitness and load‐tolerance requirements of players returning from long‐term injury.

## CONCLUSION

In this retrospective cohort of competitive football players following ACLR, high rates of RTP (84%) and second ACL injury (10%) were observed at a minimum 2‐year follow‐up. Greater exposure to OFR, particularly higher volume and weekly frequency, was associated with an increased likelihood of RTP. No association was observed between OFR compliance and overall second ACL injury risk. An exploratory subgroup analysis suggested a potential association between OFR compliance and reduced ipsilateral graft re‐rupture in players younger than 20 years; however, this finding should be interpreted with caution due to limited subgroup size.

## AUTHOR CONTRIBUTIONS

Francesco Della Villa and Matthew Buckthorpe were responsible for the conception and design of the study. Francesco Della Villa, Alberto Scavone, Daniele Caminati and Jacopo Gamberini extracted information from the patient files and performed the patient follow‐ups, establishing the patient database for the overall study design. Francesco Della Villa, Filippo Picinini and Stefano Di Paolo ran the statistical analysis and reported the results. Francesco Della Villa, Filippo Picinini and Matthew Buckthorpe wrote the manuscript with intellectual input from all other authors on subsequent drafts. Francesco Della Villa is responsible for the overall content as a guarantor.

## CONFLICT OF INTEREST STATEMENT

The authors declare no conflict of interest.

## ETHICS STATEMENT

The study received ethical approval from the Bioethical Committee of the University of Bologna (no. 0007503 of 8 January 2025).

## Supporting information

Supplementary material ‐ OFR ‐BJSM.

## Data Availability

Data are available on request.
