# Supplementary material for: The impact of on‐field rehabilitation on return to play and ACL re‐injury risk after ACL reconstruction in football (soccer) players: A study on 401 consecutive cases
Source: Knee Surg Sports Traumatol Arthrosc. 2026 Apr 14;34(6):2255–67. doi: 10.1002/ksa.70392 (PMC13266922; doi:10.1002/ksa.70392)
Supplement: Supplementary file 1 — Supplementary material ‐ OFR ‐BJSM. [file KSA-34-2255-s001.docx]

| Table 1 supplementary material. 5-stages of On-field rehabilitation, phase’s, the type of activity, and content | | |
| --- | --- | --- |
| Phase | Activity/Intensity | Progressive Sport-Specific Exercises |
| 1 | Activity without ball: Confidence acquisition toward the environment and the ground | Running in place without shoes and global coordination exercises Slow running in a straight line Back jogging in a straight line and running patterns with low-speed variations Advanced proprioceptive paths Light jumps and landings |
|  | Activity with ball | None |
|  | Cardiovascular intensity | Aerobic |
| 2 | Activity without ball: Proceed with running patterns and coordination exercises | Increasing difficulty and speed of proprioceptive paths Increasing speed of straight-line running with 'stop and go' Circular running and skip exercises Anaerobic threshold running for 8 minutes |
|  | Activity with ball | Begin to kick a soft soccer-like ball and run with it |
|  | Cardiovascular intensity | Aerobic, Anaerobic (<10% of the time) |
| 3 | Activity without ball: Running at different speeds with slow changes of direction | Slow decelerations Skips (different patterns), jumps, and landings with rotation Anaerobic threshold running for 12 minutes Technical and skill training with the ball |
|  | Activity with ball | Kicking ball exercises with lateral movements and jumps, dribbling |
|  | Cardiovascular intensity | Aerobic, Anaerobic (<30% of the time) |
| 4 | Activity without ball: Running with fast changes of direction on proprioceptive paths | Anaerobic training with sprinting and fast change of direction Decelerations Anaerobic threshold running for 16 minutes Running with faster changes of direction with the ball |
|  | Activity with ball | Technical exercises with ball from phase 3, small matches (3 vs 3, 1 vs 1), corner kicks, kick-offs |
|  | Cardiovascular intensity | Aerobic, Anaerobic (<50% of the time), Anaerobic alactacid |
| 5 | Activity without ball: Proceed with running with sprints and changes of direction | Acrobatic exercises with small obstacles High-intensity exercises Anaerobic threshold running for 20 minutes |
|  | Activity with ball | High-intensity exercises in match-like situations, maximal-intensity soccer matches (3 vs 3, 2 vs 2, 1 vs 1), power kicks, tackling |
|  | Cardiovascular intensity | Aerobic, Anaerobic (>50% of the time), Anaerobic lactacid |

**Appendix 1**

**Questionnaire for return to sport after ACL injury and rehab with Isokinetic**

| Date: |  |
| --- | --- |
| Name and Surname: |  |

Dear patient,

Thank you very much for your time and cooperation in filling out this questionnaire. Your contribution will be helpful to investigate deeper the Anterior cruciate ligament (ACL) injury, with the ambition of reducing the risk of re-injury.

**Instructions:** please, fill out the questionnaire by answering to the questions with notes or adding a “X” in the appropriate box. Please, provide answers that you think best describe the question.

**Return to sport after ACL reconstruction (ACLR)**

1. Do you feel you’re playing at **the same pre-injury level of sport** after the rehabilitation in Isokinetic?

YES

NO

Choose a number from 0 to 100:…../100


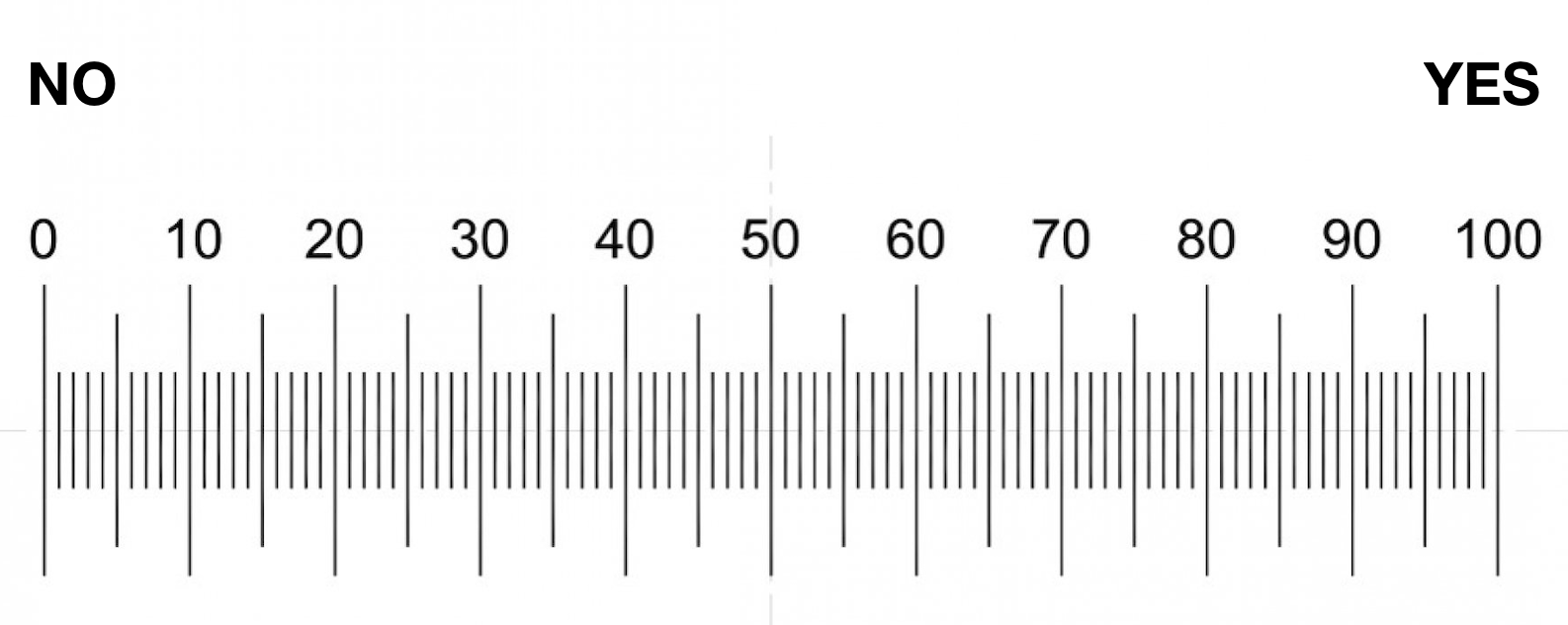


1. Did you resume playing **soccer** at competitive level after the rehabilitation with Isokinetic? *(official/organized league or at least 1 game per week)*

YES

NO

1. If you answered affirmative to question **1 and 2**, which level of soccer are you currently competing in? Please, name the league and or team you’re currently playing in:……………………………….
2. If you answered negative to question **1 and 2**, which is the main reason why you didn’t resume the same pre-injury level of competitive soccer? If the answer is not in the list please write it down here otherwise put a “X” in the box:……………………………………………………………………

“I didn’t feel physically (example: stamina, strength ext.) ready when I resumed soccer after rehabilitation” “I didn’t feel psychologically ready when I resumed soccer after rehabilitation”

“I didn’t feel my knee was recovered 100% when I resumed soccer after the rehabilitation (sometimes painful and/or I experienced discomfort when I was playing)

“When I resumed soccer, the knee was feeling great; however, I didn’t want to risk another injury”

“Other reasons NOT related to my knee (example: age, work, school or family). Please describe your answer:………………………………………………………

**Return to the team in training after the rehabilitation on the soccer pitch with Isokinetic**

1. Did you resume **training with the rest of your team** after the rehabilitation on the soccer pitch with Isokinetic?

YES

NO

1. If you answered negative to question **5**, why did you not resume training with the rest of your team? If the answer is not in the list please write it down here otherwise put a “X” in the box:……………………………………………………………………

“I didn’t feel physically (example: stamina, strength ext.) ready when I resumed team training”

“I didn’t feel psychologically ready when I resumed team training”

“I joined the team for training during a moment of the year where the team was not training (example: winter/summer break, off-season ext.)

“When I joined the rest of the team in training, after few sessions, the medical staff/manager/performance staff/ did think I was not ready enough (physically ready/performance on the pitch) to resume training with the rest of the team. Hence they separated me from the rest of the team for individual training sessions”

“I didn’t train with the team (not even for at least 1 week) because I played my first game as soon as I returned to my team for training”

1. When did you play your **first soccer match (at least 45 minutes)** after the end of the rehabilitation with Isokinetic? Please, if you remember, specify the date of the soccer match or at least the month and year: (dd/mm / 20…..).

**ACL re-injury after the rehabilitation in Isokinetic**

1. Did you sustain a second ACL injury once you resumed soccer after the rehabilitation with Isokinetic?

NO

YES, same knee

YES, other knee

1. If you answered affirmative to question **13**, when did the second ACL injury happen? Please specify the date of the second ACL injury (if you do not remember the date, please specify the month and the year) (example: 3/07/2021)

(dd/mm / yyy…..).…………………………………………..

1. If you answered affirmative to question **13**, how did the injury happen and which was **the injury mechanism** (this term describes the ACL injury causation, referring to player-to-player interaction that led to the injury)?

DIRECT CONTACT Example: “I did injured my ACL following a direct contact/collision with another player” or “I got tackled by another player and I came off the pitch as I realized I injured my ACL again”

INDIRECT CONTACT Example: “I got pushed by another player while running and following a wrong movement I got my ACL injured again” or “I jumped for a heading and in the air I hit another player and when I toke contact with the ground my knee collapsed inwards and I injured my ACL again”.

NON-CONTACT “I injured my ACL from a wrong movement I performed (example: after an intense deceleration or change of direction) and I DID NOT take any contact with other players on the pitch. I did everything on my own.

1. If you answered affirmatively to question **13, where** did the second ACL injury happen?

During a SOCCER TRAINING SESSION

During a SOCCER MATCH
